# p53-induced autophagy and senescence

**DOI:** 10.18632/oncotarget.4170

**Published:** 2015-05-19

**Authors:** Xinbing Sui, Weidong Han, Hongming Pan

**Affiliations:** Department of Medical Oncology, Sir Run Run Shaw Hospital, Zhejiang University, Hangzhou, Zhejiang, China

p53, the “cellular gatekeeper” and the “guardian of the cellular genome”, is the most commonly mutated tumor suppressor in human cancer. In response to diverse stresses (that is, DNA damage, nutrient deprivation, oncogenic activation, hypoxia, oxidative stress, hyperproliferative signals and other forms of stress), p53 can be activated and subsequently functions as a transcription factor to orchestrate various biological outputs such as transient cell cycle arrest, apoptosis, cellular senescence, differentiation, metabolism, as well as regulation of autophagy [1].

Autophagy is a dynamic catabolic process by which cytoplasmic components are targeted to a double-membrane vesicle called autophagosomes and subsequently delivered to the lysosome for terminal degradation and recycling [2]. When the cells are subjected to stressful stimuli such as starvation, autophagy is rapidly upregulated to restore their metabolic homeostasis and sustain energy requirements, thus facilitating their survival under stressful conditions. However, excessive or persistent autophagy is also shown to promote cell death. A series of stress signals can induce autophagy, including p53, nutrient deprivation, hypoxia, endoplasmic reticulum stress and so on. The direct evidence from *Scherz-Shouval et al*. suggests that p53-dependent regulation of autophagy supports cell survival in the face of chronic starvation [3]. The central checkpoints that regulate autophagy are AMPresponsive protein kinase (AMPK) and mammalian target of rapamycin (mTOR) [2].

Cellular senescence is a stress response that accompanies with the irreversible arrest and senescent morphology. Senescence may be initiated by various insults and has an important role in limiting cell proliferation, however, the mechanisms controlling cellular senescence are still debated [4]. Increasing evidence indicate that p53 and AMPK/mTOR pathway are critical mediators of the senescence response [5, 6]. Thus, p53 can induce cell cycle arrest and then active mTOR converts this arrest into senescence (geroconversion). Seemingly paradoxically, p53 can also function as a suppressor of cellular senescence by blocking the mTOR pathway [6]. Both p53 and mTOR are also involved in regulation of autophagy. mTOR inhibits autophagy. However, it remains to be determined whether p53- induced autophagy is associated with suppression of senescence (gerosuppression).

We have recently shown that p53 facilitated cell survival by inducing autophagy under the deprivation of serum [7]. Under serum starvation, an increased autophagic flux was observed in the HCT116 p53^+/+^ cells but not in the HCT116 p53^−/−^ cells, suggesting that p53 promoted autophagy under serum-starved conditions. we showed p53-dependent autophagy protected cancer cells from starvation-induced death and inhibition of autophagy by the treatment with 3-MA resulted in a large number of cell death. Thus, p53-induced autophagy functions as a survival signal in response to serum starvation.

To determine the impact of p53-induced autophagy on cellular senescence, we assessed cell cycle and staining with β-gal. We found that p53 induces cell cycle arrest and cell quiescence (not senescence) in response to serum starvation. However, β-gal activity was significantly enhanced in the serum-starved HCT116 p53^−/−^ cells, as compared with the serum-starved HCT116 p53^+/+^ cells. These results indicate that p53 may act as a suppressor of cellular senescence in response to serum starvation since β-gal is a well-known marker presented in senescent cells.

Next, we investigated whether inhibition of p53- dependent autophagy may promote cellular senescence. As mentioned above, increased autophagic flux and lack of the senescent phenotype were found in the serum-starved HCT116 p53^+/+^ cells. After the treatment with autophagic inhibitor 3-MA, the autophagy was attenuated and β-gal activity was markedly enhanced in the starved HCT116 p53^+/+^ cells, but hardly changed in the starved p53^−/−^ cells. These data indicate that inhibition of p53-dependent autophagy promotes cellular senescence. In addition, it was demonstrated that suppression of senescence through p53-dependent autophagy may occur partly through the inhibition of the mTOR pathway.

Our results demonstrate that, in the absence of serum, p53 may activate autophagic flux and subsequent autophagy functions as a survival signal to inhibit cellular senescence partly through suppression of the mTOR pathway. This study delineates a potential association between autophagy and senescence and explains why p53 may suppress cellular senescence in response to starvation. Future experiments will further illuminate the molecular mechanisms responsible for the inhibitory effects of p53-indeced autophagy on cellular senescence. A better understanding of the role of p53 in autophagy and cellular senescence will hopefully provide new strategy for cancer therapy.
